# Male or Female? For Honeybees, a Single Gene Makes All the Difference

**DOI:** 10.1371/journal.pbio.1000186

**Published:** 2009-10-20

**Authors:** Mary Hoff

**Affiliations:** Freelance Science Writer, Stillwater, Minnesota, United States of America

**Figure pbio-1000186-g001:**
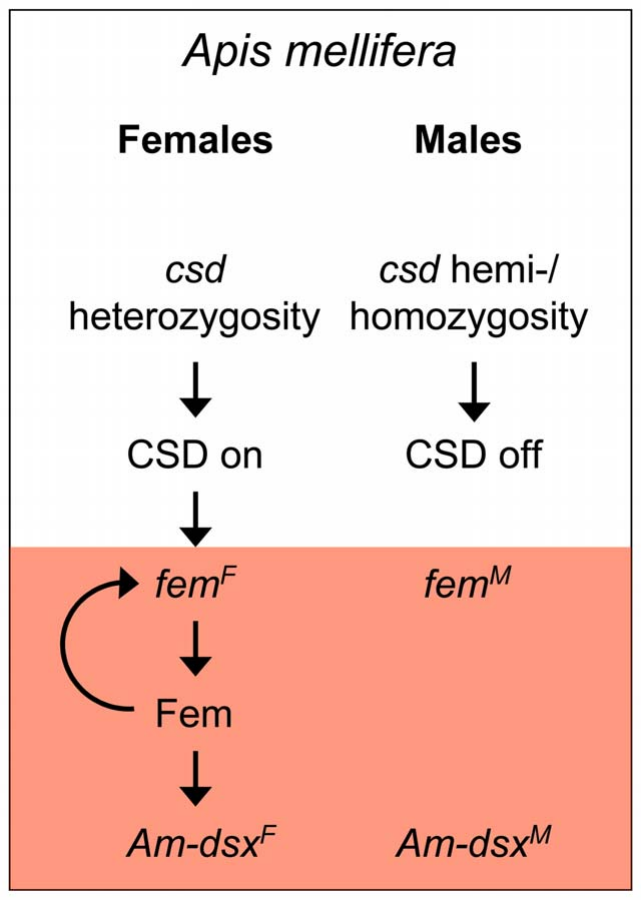
Male or female bees: It's the informational flow between genes that makes all the difference.

[Fig pbio-1000186-g001]Male or female? How genes send a developing embryo down one path or the other varies substantially among species. In honeybees, it boils down to whether a particular chromosomal location has the same version of a gene (called homozygous) or two different versions (heterozygous). Honeybees that have two different versions of the sex determination locus (SDL) develop female traits. Those that have two of the same version—or, more commonly, have only one version as a result of developing from an unfertilized egg—become male. This approach, known as complementary sex determination, is found in a number of social insects, yet is still poorly understood.

Studies of the apian approach to determining sex have identified an SDL gene, called *csd*, that is indispensable for female development when it is heterozygous. Another SDL gene required for female development, called *fem*, doesn't have to be heterozygous to do its job. In a new study, Martin Beye, Tanja Gempe, and colleagues investigate the role these genes play in determining the developmental path an embryo embarks upon and the subsequent differentiation process. Using a variety of gene-manipulating tools and very, very tiny forceps, the researchers found some surprising answers.

Before homing in on *csd* and *fem*, the researchers first asked if any other SDL genes might be involved. Well-established gene-hunting techniques confirmed the existence of three genes, besides *csd* and *fem*, in the locus—*GB11211*, *GB13727*, and *GB30480*. Repressing transcripts of these genes showed that none was involved in sex determination. In contrast, repression of *csd* or *fem* products in females resulted in development of male gonads, while repression of *csd* or *fem* products in males had no effect. Thus, *csd* and *fem* appear to be the only SDL genes involved in activating female-making machinery in honeybees.

In fruit flies, a *fem*-like gene controls somatic but not germ cell differentiation. To elucidate the extent of *fem*'s influence in honeybees, the researchers blocked transcription of *fem*. They found that the repression had no effect in males, but in females it not only induced the development of male reproductive organs (though with smaller-than-normal testes) but also caused female germ cells to differentiate into sperm rather than ova, and led development down the path to male leg morphology. When *csd* was repressed, the females transformed into males, with full-sized, normal testes. Thus, it is clear that *csd* and *fem* orchestrate the development of both somatic and germ cells.

In fruit flies, houseflies, and silk moths, *dsx*, a gene that regulates transcription of other genes, is known to influence sexual differentiation of somatic cells. Whether *dsx* steers the embryo toward maleness or femaleness depends on whether its protein products are influenced by the products of a *fem*-like gene to have a male-specific or female-specific nucleotide sequence on one end. This suggested that perhaps a similar mechanism might be operating for the honeybee version of the gene, *Am-dsx*, as well.

To test that possibility, the researchers looked at the gene transcripts, or messenger RNAs, found in pseudomale honeybees produced by repressing *csd* or *fem* in genetic females. They found protein fragments corresponding to male *fem* and *Am-dsx* when *csd* was repressed, suggesting that *csd* is the primary signal determining female differentiation and that male is the default mode. Repression of *fem* in pseudomales also resulted in male *fem* and *Am-dsx* mRNAs, indicating that *fem* is needed to induce female *Am-dsx* and that something induces *fem* activity later in development, even when initial *fem* activity is repressed.

What keeps the sex-determination machinery rolling through the rest of the development process? In fruit flies, a gene active in females encodes a protein that directs processing of that same gene's products into the female version. To test whether such a positive feedback loop might also be present in honeybees, the researchers gave males *fem*-encoding mRNA. They found the insects then produce *fem* mRNA of their own, suggesting that the *fem* is indeed part of a positive feedback loop in which its protein induces its own synthesis.

Based on these results, the researchers propose a model for honeybee sex determination in which an embryo becomes male by default unless it has two different forms of *csd*. If *csd* is heterozygous it produces an active form of the protein it codes for, which then causes *fem* to produce the female form of *fem* mRNA. The protein produced by *fem* not only causes female differentiation of somatic and germ cells, but also maintains the trajectory toward femaleness by inducing the production of the female versions of its proteins, creating a positive feedback loop in *fem*.

How exactly does heterozygosity initiate this cascade of gender-bending gene activity? The researchers hypothesize that the secret lies in binding differences in certain highly polymorphic areas of the protein product of *csd*. Heterozygosity has been shown in plants and fungi to direct development, but the mechanism involves two genes. Thus, this study appears to open the door to studies of an entirely new mechanism for gene regulation—control of a single gene via heterozygosity.

**Gempe T, Hasselmann M, Schiøtt M, Hause G, Otte M, et al. (2009) Sex Determination in Honeybees: Two Separate Mechanisms Induce and Maintain the Female Pathway. doi:10.1371/journal.pbio.1000222**

